# An Asymmetric Encryption-Based Key Distribution Method for Wireless Sensor Networks

**DOI:** 10.3390/s23146460

**Published:** 2023-07-17

**Authors:** Yuan Cheng, Yanan Liu, Zheng Zhang, Yanxiu Li

**Affiliations:** School of Network Security, Jinling Institute of Technology, Nanjing 211100, China

**Keywords:** WSN, security, key distribution, cryptography

## Abstract

Wireless sensor networks are usually applied in hostile areas where nodes can easily be monitored and captured by an adversary. Designing a key distribution scheme with high security and reliability, low hardware requirements, and moderate communication load is crucial for wireless sensor networks. To address the above objectives, we propose a new key distribution scheme based on an ECC asymmetric encryption algorithm. The two-way authentication mechanism in the proposed scheme not only prevents illegal nodes from accessing the network, but also prevents fake base stations from communicating with the nodes. The complete key distribution and key update methods ensure the security of session keys in both static and dynamic environments. The new key distribution scheme provides a significant performance improvement compared to the classical key distribution schemes for wireless sensor networks without sacrificing reliability. Simulation results show that the proposed new scheme reduces the communication load and key storage capacity, has significant advantages in terms of secure connectivity and attack resistance, and is fully applicable to wireless sensor networks.

## 1. Introduction

Wireless sensor networks (WSNs) have been proven to be suitable for large numbers of applications, ranging from industry and security domains, such as environment monitoring, fire detection and precision agriculture, to personal use, like health supervision. WSNs are composed of a large number of sensors that work independently of each other. These sensors transmit routing information to each other and forward collected application data [1,2]. The major weakness of wireless sensor networks lies in the limitations of resources, including memory, battery capacity, data processing, and communication capabilities. Sensors and wireless channels are vulnerable to eavesdropping, physical interception, malicious attacks, message tampering, identity impersonation, and side channel attacks [3,4,5], and the presence of important and sensitive information in the network increases the importance of security issues. Therefore, one of the focuses of wireless sensor network research is understanding how to provide high confidentiality for the transmitted application data and control messages to prevent various illegal attacks [6,7,8,9]. At present, it is generally believed that encryption is a key technology that can provide confidentiality between the cloud and the end [10,11,12], which can also be used in WSNs’ data exchange.

Over the years, many researchers have proposed schemes to enhance the security of wireless sensor networks. The (p, q)-Lucas polynomial-based key management scheme for WSN was proposed by Gautam et al. [13]. Their scheme outperforms other polynomials in terms of the number of keys used and efficiency. Kumar proposed a dynamic key management scheme for the clustered sensor network that supports the addition of new nodes into the network [14]. The proposed scheme has shown low energy consumption and good resiliency against node capture attacks. Moghadam et al. [15] proposed an ECDH (elliptic-curve Diffie–Hellman)-based authentication and key agreement protocol for WSN infrastructure. The proposed protocol supports the dynamic node addition in WSN environments and uses a strong ECDH technique to generate unique symmetric and session keys for each session. The authors of [16] proposed a trust-based multipath routing protocol called TBSMR, which improved the QoS and overall performance of MANETs in cellular networks through congestion control, packet loss reduction, malicious node detection, and secure data transmission. These proposals differ from the scheme proposed in this paper as TBSMR achieves power savings from the perspective of optimized routing protocols. In MANET-based medical systems, to achieve secure communication, a logic graph-based key generation scheme hybrid and encryption scheme is proposed by Sirajuddin [17], which provides high security for MANET medical networks, as well as less computational power and shorter encryption time.

In 2018, Mishra et al. proposed an authentication scheme for multimedia communications that was designed for an IoT environment base on WSNs [18]. Wu et al. [19] designed a lightweight authentication scheme for WSNs. It addressed the common security requirements and user untraceability issues. To ensure confidentiality and security in IOT, a biometric-based authentication and key agreement protocol are proposed for wireless sensor networks [20].

In recent years, researchers have produced several more viable authentication protocols and key agreements in the field of wireless sensor network security. Naresh et al. [21] proposed a lightweight multiple shared key agreement based on the hyper-elliptic-curve Diffie–Hellman method. The protocol decreases keys exchange overhead and increases the safety of the keys. In response to the security weaknesses of the scheme in [22], Shin, S. proposed a lightweight authentication based on the three-factor technique and key agreement protocol for WSN [23]. The proposed scheme addressed several security requirements and used XOR and hash functions. A lightweight password-authenticated key exchange scheme was proposed by González et al. for heterogeneous wireless sensor networks [24]. Three 3-PAKE protocols were analyzed, and the vulnerabilities of the protocols were proposed. The new protocol provided good security features with high flexibility and efficiency.

In this paper, we present a security key management scheme for cluster-based wireless sensor networks. In our scheme, session keys can be safely distributed and updated among all sensors with the help of the base station. Both static and dynamic scenarios are studied over the hierarchical networks. In particular, in our proposed scheme, the efficient encrypting algorithm makes it possible to adopt asymmetric encryption to guarantee authentication and confidentiality during data transmission.

The rest of our paper is organized as follows: Section 2 introduces security features and design constraints in WSNs; Section 3 exhibits the details of the security key management scheme; Section 4 evaluates the performance of the proposed security protocols; and Section 5 presents the conclusion and perspectives.

## 2. Design Constraints and Security Issues in WSNs

### 2.1. Physical Characteristics and Constraints

Sensors in most of wireless sensor networks are greatly limited in terms of device size, battery capacity, computing capacity, communication capacity, and storage capacity, which make the development of applications a challenge. A feasible and efficient security protocol should minimize the number of operations needed for calculation, communication, and storage. Therefore, the following characteristics of a WSN should be taken into consideration during protocol design [25,26,27,28]:

- Limited battery capacity—Sensor networks are usually deployed in outdoor environments. Due to size limitation, each sensor is usually equipped with a small battery. As a result, a sensor is unable to calculate and communicate when the battery runs out.
- Limited memory—the cache size of a sensor is usually measured in tens of megabytes, which puts forward higher requirements for the length and number of keys stored.
- Limited bandwidth—due to power limitation, most sensors use narrowband signal transmission, and the transmission rate generally does not exceed 10 KB/s.
- Limited calculation power—In order to reduce the power consumption of CPU, most sensor nodes only use 8-bit 4-megahertz microcontrollers.
- Good scalability—Wireless sensor networks must allow new legal nodes to join the existing network at any time. At the same time, the failure of any node will not affect the normal operation of the network.
- Variability in network topology—Since sensors are often installed on mobile devices, the topology of wireless sensor networks often change. Thus, network stability and nodes connectivity should be ensured in all protocol designs.
- Environment—Some wireless sensor networks are expected to be used for remote control and reconnaissance, and they are deployed in insecure and unstable environments, which makes them subject to many attacks, such as spoofing attacks, physical damage, and any other mechanical failures associated with environmental factors.

### 2.2. Security Issues in WSNs

In addition to the above characteristics of wireless sensor networks, security is also an important part of the Internet of things. Since WSNs use a wireless medium for data transmission, sensors are more vulnerable to various malicious attacks based on wireless channels. The typical malicious attacks in WSNs include eavesdropping, data modification, sink hole, spoofing attacks, denial of service attacks, sybil attacks, and node capture. For example, in node capture, the attacker accesses the hardware and software of one or more sensors through the network [29]. After successful intrusion into the sensor, the attacker steals all cryptographic keys and algorithms. Thus, it is possible for the attackers to eavesdrop and tamper with messages, as well as pretend to be legal terminals to forward data to hackers.

In recent years, a lot of research work has focused on security problems in WSNs. An asymmetric key pre-distribution scheme called AP was first proposed for hierarchical sensor networks in [30]. The famous “probabilistic” schemes had low computational complexity and communication loads. However, this scheme cannot guarantee accurate sharing of pairwise keys between any two sensors. Based on the Blom matrix, a key management scheme is proposed by Boujelben in [31] to improve the resilience against node capture. However, complex matrix operation leads to that high resource consumption by ordinary sensors. Lee presented a key renewal approach for authentication based on modular exponentiation in clustered WSNs [32]. Although this scheme improved the connectivity of the network, public-key encryption brought about a large amount of computation. Tian presented a blockchain-based trusted key management approach [33], which realized key management in WSNs through a secure cluster formation algorithm and a node mobility algorithm. In the literature [34], a novel key management model for hierarchical sensor networks based on public key infrastructure (PKI) was proposed. However, the key distribution issues in case of movement were not investigated.

### 2.3. Aasymmetric Cryptography in WSNs

Asymmetric encryption uses key pairs to encrypt and decrypt data for both sides of communication. Any message encrypted with the public key can only be decrypted by that containing the private key. The private key is secretly held by its holder, and the public key can be obtained by the required communication entity through a public channel. Asymmetric cryptography can provide confidentiality, integrity, and authentication for different kinds of networks. Although information encryption based on asymmetric key has been proved to be applicable to sensor networks, its application is still limited by its complex computation. Furthermore, taking the actual sensor chip as an example, the time taken for asymmetric encryption is still in the order of seconds, which may not be suitable for those applications with strict real-time performance.

Fortunately, in recent years, the new cryptographic algorithms have shown great energy efficiency and reached the same security level as traditional algorithms. For example, the elliptic-curve cryptography (ECC) [35] method is the representative version of those algorithms. ECC is a cryptographic regime built on the discrete logarithm problem of elliptic curves. Using point *G* on an elliptic curve and integer *k*, it is easy to find *K* = *kG*. Conversely, using the points *K* and *G* on an elliptic curve, finding the integer *k* is a difficult task. The main advantage of ECC is that it uses smaller keys and provides a considerably higher level of security. The 164-bit key in the ECC algorithm can provide a level of security equivalent to the strength of secrecy provided by the 1024-bit key in the RSA algorithm. The ECC algorithm is less computationally intensive, is faster to process, and takes up less storage space and transmission bandwidth. Therefore, Bitcoin has also chosen ECC as its encryption algorithm.

In [36], the author proposed a new SUA-WSN scheme based on elliptic-curve cryptography (ECC) and proved that it achieves user anonymity, as well as AKE security, in the extended model. Gulen et al. implemented ECC on the MSP430 microcontroller, which is a widely used microcontroller in WSNs, using Edwards curves for point arithmetic and the number theoretic transform for the underlying finite-field multiplication and squaring operations [37]. Gulen’s research shows better timing values and can be applied to ECC implementations.

From the perspective of energy consumption and computational complexity, ECC has promising uses for data encryption in WSNs. It provides comparative security with a smaller key, which also reduces the energy of computation and communication in WSNs. Based on this method, a new security key management scheme and an authentication approach are proposed in Section 3.

## 3. The Key Management Scheme for Cluster-Based WSNs

In this section, a security key management scheme for wireless sensor networks based on public-key cryptography is presented. To avoid long-term attacks through which attackers can analyze the encrypted traffic over the network for a long period of time, a key update approach is specifically designed.

### 3.1. Network Model and Assumptions

At present, wireless sensor networks commonly used in the industry mainly include two kinds of architectures, namely hierarchical structure and flat structure. A hierarchical architecture is usually used for large-scale WSNs due to its good scalability. A clustered hierarchical network is composed of base stations (BS), a large number of sensor nodes, and a small number of cluster heads (CH). BS is not limited by resources. The base station is responsible for managing all nodes of the network and receiving the service data collected via the sensor nodes. It is assumed that the cluster head has a higher configuration than the sensors, including battery capacity, memory size, communication, and computing capacity. Like the gateway, the cluster head assists in data transmission between the sensors and the base station. In the hierarchical architecture, sensors are divided into non-overlapping clusters, which collect data from the surrounding environment and send the original data to the base station. In this article, we focus on hierarchical architecture of WSNs.

In our scheme, asymmetric encryption is used to realize the authentication between the base station, the CHs, and the sensor nodes. The public key is pre-loaded into each sensor before network deployment. With the public-key system, the proposed scheme not only realizes end-to-end identity authentication, but also provides security for subsequent key distribution processes.

In our hierarchical WSN model, we make the following few assumptions:

- The base station has more energy power for calculations and communications than sensors.
- The base station owns a pair of keys (a public key and a private key).
- The network is divided into several cluster regions. In each cluster, there is only one cluster head node, and its location remains unchanged. Each cluster head can be recognized as the gateway of its cluster.
- In terms of security and ease of management, each cluster generates different session keys for dialogs between sensor nodes and cluster heads.
- Both asymmetric and symmetric cryptography are used for each sensor. The former method provides mutual authentication and key distribution, and the latter method preserves the confidentiality of traffic transmitted.
- As an optional technology in our scheme, MAC (message authentication code) provides data integrity.
- The public key is pre-loaded into each sensor and the cluster head via an off-line dealer.
- Each sensor can store at least one public key and several session keys in its memory.
- Each sensor can randomly move among different clusters at a low speed.

### 3.2. Network Initializtion and Definitions

**Figure 1:**
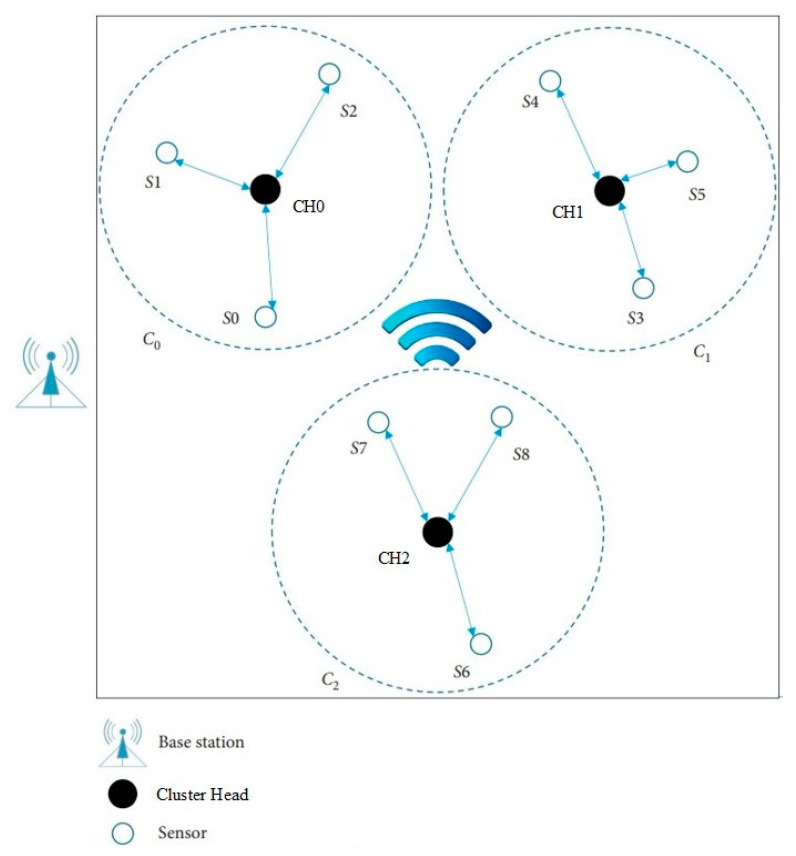
The network topology.

In the network, there are *n* sensors, which are denoted as *S*_0,…,*n*−1_, and *m* cluster heads (CH), which are denoted as CH_0,…,*m*−1_. Each sensor has a unique identification code *ID_si*, which has a length of 2 bytes stored in the chip. After the initialization of the network is completed, all nodes automatically run the cluster formation algorithm (this algorithm is not discussed in this paper; for more information, please refer to [38]), which results in *m* clusters being formed randomly by all nodes. There is only one CH and *n*/*m* sensor in each cluster. Figure 1 shows a typical network of three clusters. Each cluster contains one CH and three sensors.

After network deployment, each CH runs a cluster forming process, and sensors are divided into clusters with no cross coverage. After a period of operation, some sensor may move into another cluster’s region. In this situation, the subsequent key distribution and update process will be performed via the CH of the present cluster. In the following section, we will describe the scheme in regard to two aspects: static sensors and mobile sensors.

The following definitions will be used in our scheme and analysis:

*SK_i_* denotes the symmetric session key with a length of 16 bytes shared by the base station and sensors located in *DG_i_*.

*PUK* denotes the public key of the BS, and *PVK* denotes the corresponding private key. *PUK* can be obtained through public key infrastructure (PKI).

The function *E*(*x*,*y*) denotes encryption (symmetric or asymmetric) operation, parameter *x* denotes encryption key, and parameter *y* denotes the plain message that needs to be encrypted. The function *D*(*x*,*y*) denotes decryption operation.

*ID__CHi_* denotes the identity code of the cluster with a length of 1 byte, and it can be acquired using the CH of that cluster. It is stored in the chip of each CH, and a tamper proof mechanism is used.

*ID_si* denotes the identity code of sensor *S_i_* up to a maximum length of 2 bytes. It is stored in the chip of each sensor, and the tamper proof mechanism is used.

### 3.3. Static Sensors Subscheme for Hierarchical WSNs

#### 3.3.1. Mutual Authentication and Key Distribution Process

In our clustered architecture network, the CH plays an important role in the process of key management. The key problem here is understanding how to distribute the key among the sensor nodes under many restrictions. We assume that all sensors are static and present the operations of handshake, key distribution, authentication, and key update. The handshake is destined to establish a symmetric key shared by sensors and BS. The operation of handshake includes three steps:

1. **Generation of the *SK_i_*:** The *CH_i_* generates a random symmetric key *SK_i_* and a challenge *R*. Next, the *CH_i_* encrypts *SK_i_*, *R*, and *ID__CHi_* with *PUK*, and we find
  Cipher1 = *E*(*PUK*, *SK_i_*‖*R*‖*ID__CHi_*‖*timestamp*) (1)
  The 2-byte timestamp is used to resist replay attacks. *CH_i_* sends Cipher1 to the base station using traditional routing. Here, the *PUK* is used for authentication and preserving the confidentiality of the session key *SK_i_*.
2. **Establishment of *SK_i_*:** After receiving and decrypting the message, the base station finds *SK_i_*, and *R* uses its *PVK* and builds a global table of all session keys of different clusters. This table is used to identify the cluster and its cluster head on the network. Meanwhile, if *ID__Chi_* can be found in the database of legal CHs, the identity of the *CH_i_* can be authenticated using BS.
3. **Completion of the handshake:** The base station encrypts *R* with the established session key *SK_i_*. and finds
  Cipher2 = *E*(*SK_i_*, *R*) (2)

Next, the base station sends Cipher2 to *CH_i_*, and *CH_i_* decrypts it. When the challenge *R* is correctly received, a session key is successfully established between BS and *CH_i_*. Otherwise, *CH_i_* will reinitiate the handshake. Considering the resource consumption caused by the computational complexity, the message authentication code (MAC) is not added to the key distribution process.

Through the above steps, the mutual authentication between the base station and *CH_i_* is completed. After that step, each sensor in the cluster needs to achieve the session key *SK_i_* generated using *CH_i_*. Thus, sensor node *S_i_* builds a message encrypted using the *PUK*, which is denoted as follows:

Cipher3 = *E*(*PUK*, *ID__CHi_*‖*ID_si*‖*timestamp*‖*SK_si*‖*R*) (3)

where *SK*_*si* is a symmetric key generated using sensor *S_i_*. For sensor *S_i_*, the Cipher3 is used to apply for the session key and identity authentication at the same time.

When the BS receives Cipher3, it picks out the corresponding session key *SK*_*si* according to *ID__CHi_*. At the same time, if the *ID_si* can be found in the list of legal sensor nodes, the authentication of *S_i_* is also accomplished.

To secure the session key, the base station encrypts *SK_i_* with the session key *SK*_*si* and builds the Cipher4 as follows:

Cipher4 = *E*(*SK*_*si*, *SK_i_*‖*R*). (4)

**Figure 2:**
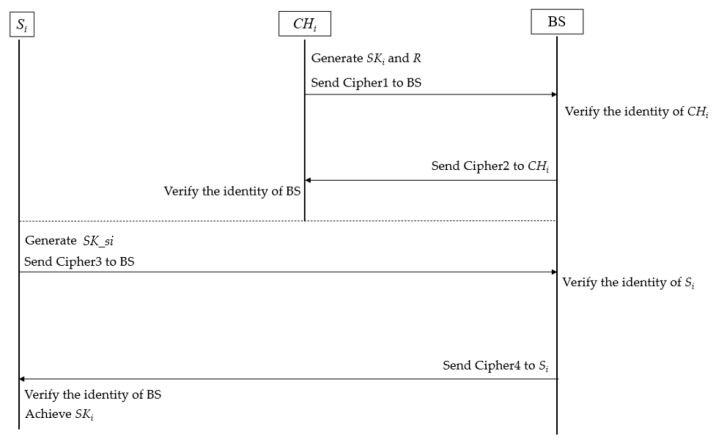
Flowchart of authentication and key agreement in the static scenario.

Next, the Cipher4 is sent to *S_i_*, and *S_i_* will decrypt it using the symmetric key *SK*_*si*. Finally, all sensors in the same cluster have the same session key *SK_i_* as its cluster head. Through the above key distribution subscheme, the confidentiality of traffic between the cluster head and the sensor is guaranteed. Moreover, mutual authentication between the BS and *S_i_* is successfully performed. The detailed key distribution process is depicted in Figure 2.

**Figure 3:**
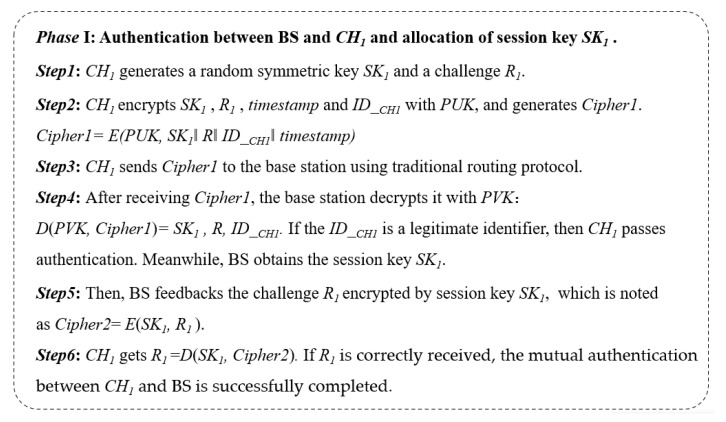
Specific steps for phase I in an example.

**Figure 4:**
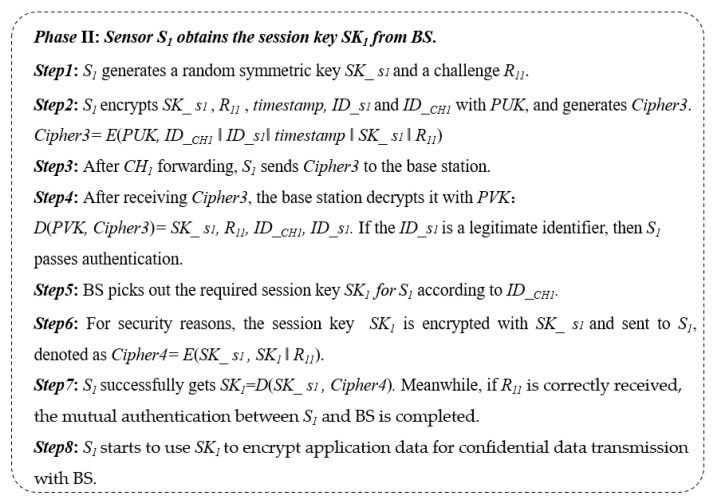
Specific steps for phase II in an example.

The specific implementation process of our proposed asymmetric encryption-based key distribution method in the static scenario is shown in Figure 3 and Figure 4. In phase I, CH_1_ and BS complete the two-way authentication and distribution of the session key *SK*_1_ at the same time. In phase 2, the secure distribution of the session key between sensor *S*_1_ and BS is realized.

#### 3.3.2. Session Key Update Process

To protect the nodes against long-term attacks, a periodic key update mechanism is designed. The steps of the key update are given as follows.

1. The new session key *SK_i_*’ is generated via the cluster head *CH_i_* at a certain moment.
2. *CH_i_* notifies the base station to update the session key.
3. Using the proposed handshake operation, the new session key *SK_i_*’ is distributed between the BS and the *CH_i_*. After that step, the *CH_i_* notifies all sensors to update their session key in its cluster with a broadcasting message. Sensors will stop encrypting sessions until they receive the new session key *SK_i_*’.
4. After the establishment of *SK_i_*’, the *CH_i_* distributes *SK_i_*’ encrypted using the original session key *SK_i_* to all sensors by broadcasting cipher5, which is denoted as follows:
  Cipher5 = *E*(*SK_i_*, *SK_i_*’). (5)
5. Each sensor in the cluster decrypts the cipher5 using the old session key *SK_i_* and substitutes it for the *SK_i_*’. The subsequent dialog is decrypted using the new session key.

### 3.4. Mobile Sensors Subscheme for Hierarchical WSNs

#### 3.4.1. Mutual Authentication and Key Distribution Process

**Figure 5:**
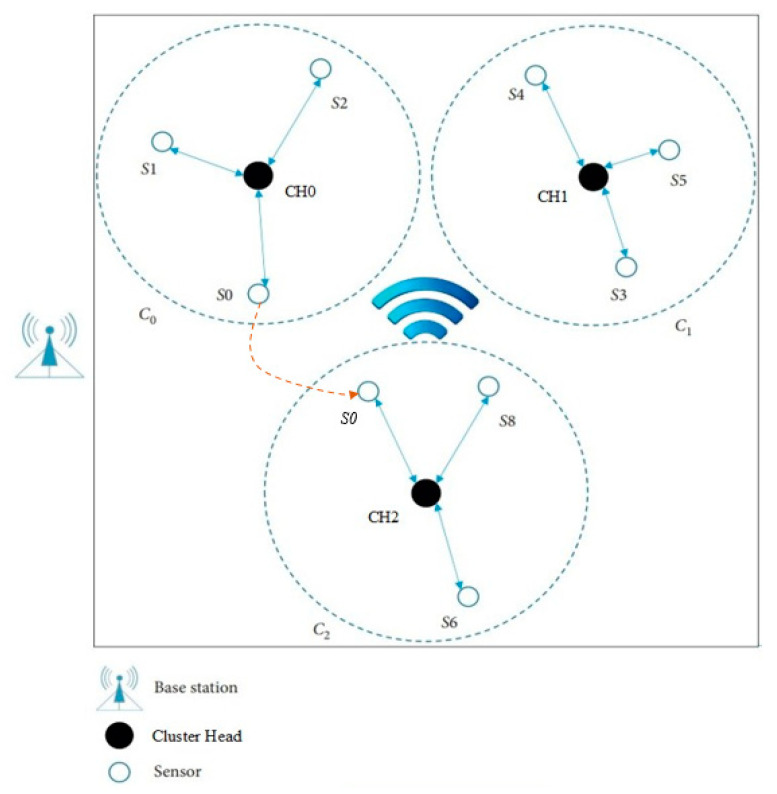
Sensor *S*_0_ moves from Cluster0 to Cluster2.

Since sensor nodes have a high probability of moving between different clusters of the network, the dynamic subscheme for hierarchical architecture is more complicated. In Figure 5, *S*_0_ moves from the cluster *C*_0_ into another cluster named *C*_2_. As the location of each CH is assumed to be unchanged, the process of authentication and key distribution between CH and BS is the same as that of the static subscheme. The main difference between the static subscheme and the mobile subscheme lies in the key distribution process.

The key distribution process of the mobile scene includes six steps.

1. When *S*_0_ moves into cluster2, it will send a cluster-entry request to CH_2_. The cluster forming and cluster head detection process is not described in this paper. For more information, please refer to [24].
2. CH_2_ detects and receives this message. Next, CH_2_ replies to *S*_0_ with a message including its identification code *ID__CH_*_2_.
3. *S*_0_ updates the identification of the present cluster, replacing *ID__CH_*_0_ with *ID__CH_*_2_.
4. *S*_0_ applies for the latest session key *SK*_2_ via the base station using the cipher6 denoted as follows:
  Cipher6 = *E*(*PUK*, *ID_*_*CH*2_‖*ID_*_*S*0_‖*timestamp*‖*SK_*_*S*0_‖*R*) (6)
5. The BS decrypts cipher6 with the *PVK* and finds *ID__CH_*_2_, *SK__S_*_0_, and *ID__S_*_0_ via
  Plain6 = *D*(*PVK,* Cipher6) = *D*(*PVK*, *E*(*PUK*, *ID__CH_*_2_*‖ID__S_*_0_*‖timestamp‖SK__S_*_0_‖*R*)) = *ID__CH_*_2_*‖ID__S_*_0_*‖SK__S_*_0_‖*R.*
  The latest session key *SK*_2_ can be picked out in terms of *ID__CH_*_2_, and the *S*_0_ is authenticated via BS according to *ID__S_*_0_. Next, the cipher7 will be sent to *S*_0_. The cipher7 is built as follows:
  Cipher7 = *E*(*SK__S_*_0_, *SK*_2_‖*R*). (7)
6. *S*_0_ decrypts the cipher7 with the symmetric key *SK__S_*_0_ and successfully finds *SK*_2_.

**Figure 6:**
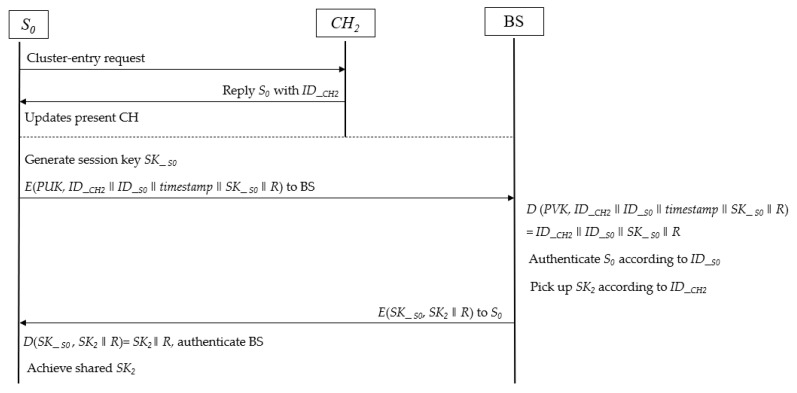
Flowchart of authentication and key agreement in the mobile subscheme.

Thus, the mobile sensor can achieve the latest session key of the present cluster and send encrypted traffic to the corresponding cluster head. The detailed key agreement process in mobile subscheme is depicted in Figure 6.

#### 3.4.2. Session Key Update Process

However, when *S*_0_ moves to the junction of two adjacent clusters, for example *C*_0_ and *C*_2_ in Figure 5, it may receive key update messages from CH_0_ and CH_2_ at the same time. It should be noted that *S*_0_ only knows the previous session key *SK*_0_ of cluster0, and it is unaware of the previous session key of cluster2. Thus, *S*_0_ can only decrypt the broadcasting message from CH_0_ to update *SK*_0_. After joining cluster2, *S*_0_ can obtain the present session key *SK*_2_ from the base station and wait for key updating to repeat.

## 4. Analysis and Comparison

Extensive simulations are provided to verify the performance of our scheme, such as memory consumption, communication overhead, connectivity, and recovery capability for node capture. Next, we compare the proposed key management scheme with other schemes from multiple dimensions.

We evaluate the performance based on NS-2 [39]. In the simulation, we randomly arranged a total of 200 sensors and 20 cluster head nodes with dimensions of 100 m by 100 m. Each sensor moves at a speed of 1–5 m/s. The signal reception range of each sensor is 10 m. The data transmission rate is 32 kbps; the traffic generation uses the CBR model, and the traffic generation interval is 30 s.

### 4.1. Key Storage of Sensor Nodes

In our scheme, the public key is pre-loaded into sensor’s memory during the network initialization. Since the strength of encryption with the 256-bit ECC key is equal to that of the 3072-bit RSA key, a public key of 256 bits in length is used in our simulation. Moreover, two 16-byte session keys are used in the key distribution process. When a sensor receives the refreshed session key, the original key will be deleted to save the memory. Therefore, the memory overhead of each sensor is only 64 bytes, while that of the CH is 48 bytes.

The key distribution in [30] is that *k* keys are pre-loaded into each sensor, while *m* keys (*m* ≫ *k*) are pre-loaded into each CH. If any two nodes share a pairing key, they can establish a secure link. Thus, the greater the number of keys stored, the higher probability of sharing common keys. In [40], the memory is divided into two parts. One part is used to store *α* pre-distributed keys, and the other part is used to store *β* post-deployment keys.

Table 1 presents the key storage overheads in different schemes. For large- and medium-sized wireless sensor networks, sensors in our scheme require less storage space than those of other schemes. However, our cluster heads require slightly more memory space than those of Erfani’s scheme. Since the number of sensors is much larger than that of CHs, our scheme is valuable for resource-limited WSNs.

### 4.2. Communication Overhead

The communication overhead in our analysis only considers the payload related to key distribution and update, and it does not include the IP packet encapsulation of the network layer.

The length of AES-based session key is set to 16 bytes. The bytes of IP message encapsulation are not included in the calculation of the traffic generated during key distribution and update. For the static scenario, in stage 1, the effective communication load between the cluster head and the base station is 32 bytes. In stage 2, the effective communication load between the sensor node and the base station is 64 bytes. Therefore, the communication load consumed by a cluster for a complete key distribution process is 96 bytes. In the key update phase, the effective communication load between the cluster head node and the base station and the sensor nodes is 64 bytes in total, of which the load of broadcasting messages to the sensors in the cluster makes up 32 bytes. As for the dynamic scenario, the communication overhead of the CH and the sensor are the same as that of the static scenario.

As the frequency of session key update increases, the bandwidth occupied by key distribution also increases. This outcome means there is a tradeoff between security and communication load in wireless sensor networks.

### 4.3. Security Analysis

#### 4.3.1. Mutual Authentication

In both subschemes, mutual authentication of BS and sensors (including CHs) is assured via the challenge–response mechanism. Terminals without legal identifiers (*ID__CHi_* or *ID_si*) cannot pass the identity authentication. Since the identifier is stored in the chip of each sensor with a tamper proof mechanism and encrypted for transmission, its confidentiality and integrity can be guaranteed. We added 10 nodes to the test network and distributed them evenly in 3 clusters. They simulated nodes that gained illegal access to the sensing network, randomly generating their identification codes *ID_si*. Since the identifiers *ID_si* used by these 10 nodes in constructing the *Ciperh3* were not included in the authorized and legitimate user list of the base station, the shared session key could not be obtained via the base station in the test. As a result, the reliability of the authentication scheme is fully demonstrated.

#### 4.3.2. Security Connectivity

The security connectivity is defined as the probability that two nodes successfully establish a session key. Since authentication and key distribution in our proposal are cluster based, we define “inter-cluster connectivity” as the probability that a CH shares a pairwise key with the sensors in its cluster.

**Figure 7:**
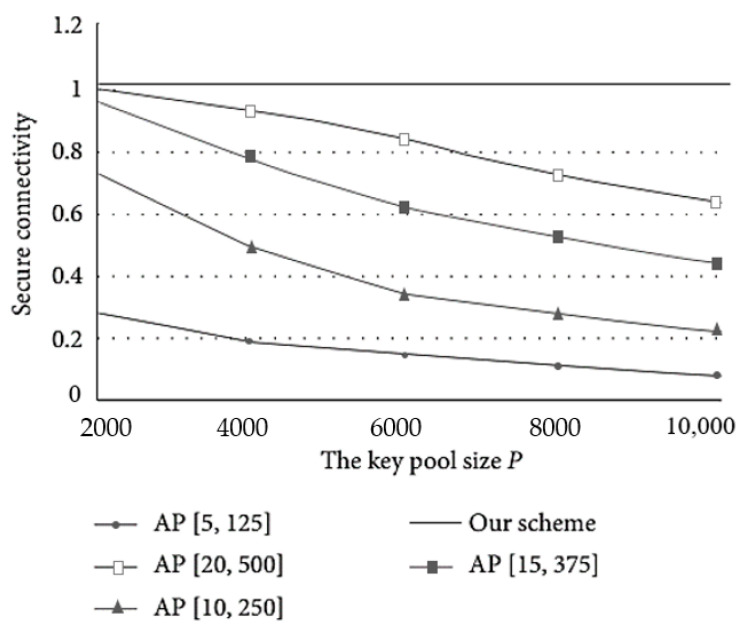
Secure connectivity versus key pool size *P*.

In our deterministic key distribution scheme, each authenticated sensor can always successfully share a session key with the present cluster head. Compared to the probabilistic key distribution approaches in [30,31,41], the inter-cluster connectivity in our scheme is 100%. Those random schemes, like AP [30], can only achieve higher security connectivity by increasing the amount of key storage. Figure 7 depicts the comparison of secure connectivity and key pool size in the AP. As the number of pre-loaded keys increases, the performance of the secure connectivity gradually improves. For fixed parameters [*l*, *M*], the security connectivity decreases significantly as the key pool increases.

#### 4.3.3. Resistance to Attacks

The new scheme provides a set of session keys to secure data exchange between the base station and sensors. Our proposal, which is based on session and public keys, can effectively resist common network attacks.

Eavesdropping can be avoided using symmetric encryption, as well as the key update mechanism proposed in this article. Spoofing attacks are avoided in our scheme through mutual authentication based on public-key encryption. Moreover, the authenticity of sensors is achieved via a challenge–response mechanism, and the identity code is preloaded before deployment.

Attacks like modification, reply, and insertion can be resisted via symmetric encryption and message authentication code added to each message. Only those authenticated nodes can send or modify data packets on the network.

Attackers obtain the secret information by capturing nodes or other physical means. We define resilience against node capture as the probability *F*(*x*) that attackers obtain the key from the uncaptured node according to a certain number of captured nodes *x*. Thus, we find

(8) $$F\left(x\right)=\frac{\mathrm{number}\;\mathrm{of}\;\mathrm{compromised}\;\mathrm{links}\;\mathrm{between}\;\mathrm{uncaptured}\;\mathrm{nodes}}{\mathrm{number}\;\mathrm{of}\;\mathrm{uncompromised}\;\mathrm{links}}$$

**Figure 8:**
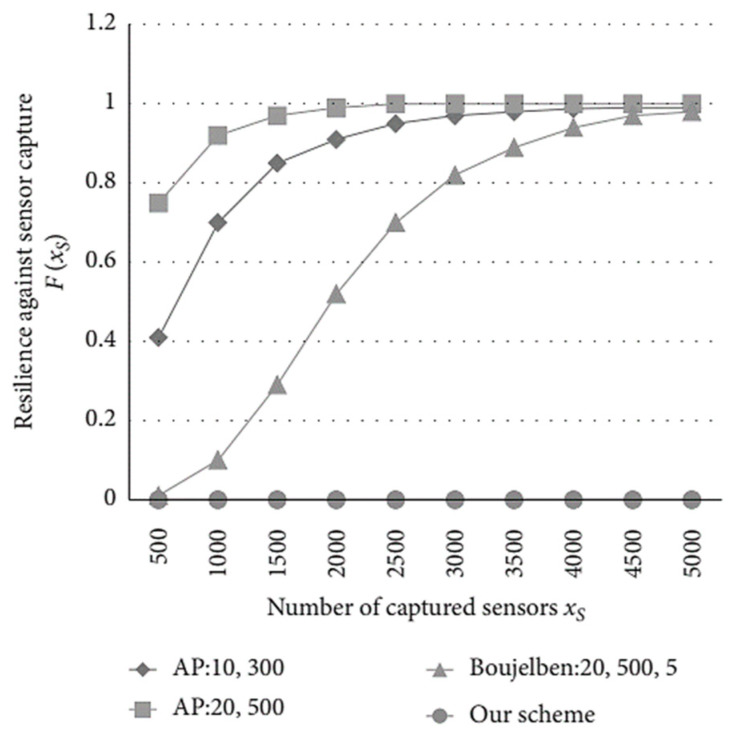
The probability of resilience against sensor capture in different schemes.

Resilience against sensor capture is first evaluated. Unlike the random key pre-distribution schemes in [10,11,42], sensors only need to pre-load a public key in our approach, which saves the memory of the sensor node. Due to the periodical key update applied, it is too hard for attackers to find the constantly updated session key, despite physically capturing a sensor in our proposal. Thus, the probability of resilience against node capture is *F*(*x_s_*) = 0, where *x_s_* represents the number of captured sensor nodes. As shown in Figure 8, the resilience performance worsens with the increasing number of captured nodes for random key pre-distribution schemes, because of the storage of a large number of session keys. Since the sensors store matrixes instead of keys, the resilience performance of Boujelben’s scheme [31] is better than that of the AP scheme [30]. Simulation results indicate that threat of sensor capture is perfectly eliminated via our scheme.

Finally, Table 2 presents several typical schemes of key management in WSN that emerged recent years. In our scheme, we provide a simple and feasible mutual authentication mechanism comparable to [30,34,40]. Lee, in [32], used an asymmetric encryption algorithm with more computation overhead than in [34] and our proposal. Furthermore, our scheme outperforms other schemes in terms of resilience against node capture and resistance to eavesdropping.

## 5. Conclusions

The research work discussed in this paper focuses on key distribution schemes for static and dynamic wireless sensor networks. The novelty of this scheme is that the proposed key distribution and update strategy is particularly suitable for sensing networks in which the nodes are in motion. In addition, we evaluate the design scheme in terms of key storage capacity and the communication load generated during key exchange and security. Compared to the traditional classical key distribution scheme, our proposed new scheme is less complex to implement, reduces the cache capacity requirements of the nodes, and obtains better connection security and resistance to attacks. It can be concluded that our results are particularly suitable for wireless mobile sensing networks with high capacity, low power consumption, and high reliability requirements, such as environmental monitoring networks, energy IoT networks, and smart warehouse management systems.

## Figures and Tables

**Table 1:** Key storage overheads (bytes) in different schemes.

	Du [30]	Erfani [40]	Our Scheme
Sensor	32*l*	32 (α and β)	64
Cluster Head	32*M*	32	48

**Table 2:** Security comparisons of different key distribution solutions.

Scheme Features	Du [30]	Lee [32]	Benamar [34]	Erfani [40]	Our Scheme
Public-key encryption	—	√	√	—	√
Key pre-distribution	√	×	√	√	√
Mobility of sensors	—	×	×	√	√
Perfect resilience against node capture	×	—	—	×	√
Mutual authentication	×	√	×	×	√
Resistant to eavesdropping attacks	—	—	√	√	√

—: Not involved. √: Support. ×: Not Support.

## Data Availability

Not applicable.
